# The Role of Innovation in Delivering the 2021–2030 Neglected Tropical Disease Road Map

**DOI:** 10.4269/ajtmh.21-1178

**Published:** 2022-03-15

**Authors:** Mwelecele Ntuli Malecela

**Affiliations:** World Health Organization, Geneva, Switzerland

Two years ago, with the support of the neglected tropical diseases (NTDs) community, World Health Organization developed a new high-level road map, *Ending the neglect to attain the Sustainable Development Goals: a road map for neglected tropical diseases 2021–2030.* This document supplants the previous road map, which defined work and progress to 2020 and drove some vital achievements, with unprecedented progress in controlling and eliminating many NTDs—notably sleeping sickness, Guinea worm disease, lymphatic filariasis, and trachoma. It was also the guiding document in force as the global NTD community made several major technical leaps forward, including the development, from conception to implementation, of the use of ivermectin, diethylcarbamazine, plus albendazole (IDA), in combination, for lymphatic filariasis, which has laid the groundwork for accelerated elimination of this disease in many countries. Despite these and many other successes, however, it became obvious that not all of the 2020 targets would be met, and this led to a period of reflection and reevaluation.

As part of this process, WHO undertook a comprehensive consultative process, and I am proud to say that we at WHO did a good deal more listening than talking. We heard about what mattered most to our partners including, crucially, the health ministry teams who work so hard to prevent and treat these diverse diseases. Their responses focused on areas such as integration, collaboration, and partnership. Time and again, we heard also that talking about diseases themselves was nowhere near as important as talking about the people who suffer their effects.

We heard that countries wanted to take ownership of their duty of care to communities and populations, and that they wanted the rest of us, as a global NTD community, to support them in that endeavor. Our listening exercise resulted eventually in a flagship road map document, one which has at its core a desire to align and enable the success of the United Nations sustainable development goals and to deliver equitable care for all.

Then, of course, COVID-19 arrived, a pandemic the like of which none of us has seen and which has affected every country on earth and amplified existing inequity. The reach of COVID-19 across the globe, and the necessity to divert resources to combat this emergency, has had an impact on every part of the work we do and every link in the chain.

That said, not all of the impacts of COVID-19 have been negative—and one field in which this is particularly true is innovation. If innovation can be described as any practice that leverages creative invention to respond to an important challenge, it seems evident to me that the stimulus toward innovation is greater and the resulting response more vigorous now than ever before. For example, the need for accurate testing and for vaccines for COVID-19 has led to innovation on a scale and in a timeframe that was previously unheard of. It was also evident that end-to-end processes could be expedited. We have seen this before in NTDs, particularly, of course, with IDA for lymphatic filariasis, the subject of this Supplement. In that case, the translation of research into implementation was streamlined and rapid. The potential for using such highly efficient approaches in the NTD field is very exciting. It is also critical in terms of achieving the 2030 goals.

Now, as we look forward to an eventual post-COVID-19 landscape, we must presume that there will be fewer resources available to us than perhaps has been the case in the past. The demands made on these resources will not reduce, however. In fact, they will only increase. It is vital, therefore, that we heed the lessons of COVID-19, and in particular the clarion call for greater and more far-reaching innovation. Inadequate diagnostics remain a huge impediment to our progress toward the 2030 road map targets, as does the paucity of options in the therapeutic arsenal. A key question for our domain revolves around leveraging creative inventions to respond to the challenges we have before us. Do we have the investments required not only to find innovative products and strategies, but also to apply those innovations where they are most needed?

Our work to combat NTDs must necessarily be carried out in conjunction with many other programs—malaria, TB, immunization, and more. All of these programs are themselves facing up to a more uncertain world and their own needs for innovation. Failure to collaborate and pool resources between fields and specific interests will jeopardize all the progress we have made, collectively, over the last decade.

COVID-19 has also, to my mind, made our new NTD road map even more relevant than it was at its inception. At its heart, it seeks to promote resilience, health system-strengthening, equity, and country ownership. None of these goals can be imposed; they require, rather, that we collaborate. I am more certain than ever that these principles will enable us not only to control, to eliminate, and to eradicate specific NTDs, but also to demonstrate through our collective action that health is a basic and universal human right, regardless of social, economic, or national status. I am as optimistic as always and look forward to working with the scientists contributing to this Supplement, along with many others, to deliver the benefits of innovative approaches to NTDs to the patients and populations that need them the most.

